# Limitation of cellulose accessibility and unproductive binding of cellulases by pretreated sugarcane bagasse lignin

**DOI:** 10.1186/s13068-017-0860-7

**Published:** 2017-07-11

**Authors:** Germano Siqueira, Valdeir Arantes, Jack N. Saddler, André Ferraz, Adriane M. F. Milagres

**Affiliations:** 1 Departamento de Biotecnologia, Escola de Engenharia de Lorena, Universidade de São Paulo, CP 116, Lorena, SP 12602-810 Brazil; 2 Forest Products Biotechnology/Bioenergy Group, Faculty of Forestry, University of British Columbia, 2424 Main Mall, Vancouver, BC V6T 1Z4 Canada

**Keywords:** Sugarcane bagasse, Thermochemical pretreatment, Lignin removal, Cellulose accessibility, Unproductive binding, Protein binding capacity

## Abstract

**Background:**

The effectiveness of the enzymatic hydrolysis of cellulose in plant cell wall is strongly influenced by the access of enzymes to cellulose, which is at least in part limited by the presence of lignin. Although physicochemical treatments preceding the enzymatic catalysis significantly overcome this recalcitrance, the residual lignin can still play a role in the process. Lignin is suggested to act as a barrier, hindering cellulose and limiting the access of the enzymes. It can also unspecifically bind cellulases, reducing the amount of enzymes available to act on cellulose. However, the limiting role of the lignin present in pretreated sugarcane bagasses has not been fully understood yet.

**Results:**

A set of sugarcane bagasses pretreated by five leading pretreatment technologies was created and used to assess their accessibility and the unproductive binding capacity of the resulting lignins. Steam explosion and alkaline sulfite pretreatments resulted in more accessible substrates, with approximately 90% of the cellulose hydrolyzed using high enzyme loadings. Enzymatic hydrolysis of alkaline-treated (NaOH) and steam-exploded sugarcane bagasses were strongly affected by unproductive binding at the lowest enzyme loading tested. Analysis of the extracted lignins confirmed the superior binding capacity of these lignins. Sulfite-based pretreatments (alkaline sulfite and acid sulfite) resulted in lignins with lower binding capacities compared to the analogue pretreatments without sulfite (alkaline and acidic). Strong acid groups present in sulfite-based pretreated substrates, attributed to sulfonated lignins, corroborated the lower binding capacities of the lignin present in these substrates. A more advanced enzyme preparation (Cellic CTec3) was shown to be less affected by unproductive binding at low enzyme loading.

**Conclusions:**

Pretreatments that increase the accessibility and modify the lignin are necessary in order to decrease the protein binding capacity. The search for the called weak lignin-binding enzymes is of major importance if hydrolysis with low enzyme loadings is the goal for economically viable processes.

## Background

The hierarchical organization of three main components of the composite-like structure of the plant cell walls is essential for plant development and growth. Cellulose is the major constituent and corresponds to approximately 50% of cell wall dry mass. The other components, hemicellulose, and lignin complete the resistant structure and give cell wall other vital characteristics, such as partial flexibility and hydrophobicity [1, 2]. Cellulose being the most abundant natural polymer on Earth has been the spotlight of several industrial processes either as a polymeric structure (i.e., cellulosic pulp, cellulose derivatives, and nanocellulose) or as a source of glucose for fuels and chemicals production. For cellulose depolymerization, enzymatic catalysis using cellulases and accessory enzymes has been explored over the past decades and is already available in commercial plants, but the process is still not completely understood [3].

Because of the strong interaction of components, plant cell walls are highly recalcitrant and the utilization of their components in the biorefinery concept requires cell wall fractionation, at least partially. This step, known as pretreatment, is crucial to reduce cell wall recalcitrance and enhance cellulose hydrolysis [4]. Despite the many variables in a pretreatment, most of them are based on reactions either in acidic or alkaline conditions. In brief, pretreatments in acidic conditions aim hemicellulose solubilization, leaving a pretreated solid rich in cellulose and lignin. The liquid fraction containing mostly hemicellulose monomeric (or oligomeric, depending on the pretreatment conditions) sugars can be fermented to produce ethanol or other products [5–7]. The cellulose in the solid fraction can be enzymatically hydrolyzed with higher yields than untreated feedstock, mainly due to increased accessibility (hemicellulose removal and lignin relocation) [8–11]. As a matter of fact, most of the cellulosic ethanol plants around the world are based in acid pretreatment technologies [12]. Alkaline pretreatments are more efficient in lignin removal, substantially increasing cellulose digestibility, even after removing only part of the lignin [13–15]. Since most of the hemicellulose remains in the solid fraction, accessory enzymes (xylanases, esterases, and some oxidases) are necessary in the enzyme mixture [16, 17].

In both acidic and alkaline conditions, lignin solubilization can be enhanced by the presence of nucleophiles such as sulfite ions [14, 18–20]. In acid conditions, a sulfite-based pretreatment was developed in order to remove lignin and achieve better cellulose conversion yields [19, 21]. However, even under conditions in which lignin removal is favored, the residual lignin is known to play a limiting role in the enzymatic hydrolysis of the cellulose. It has been reported that the residual lignin can act as a barrier, hindering the access of cellulolytic enzymes to cellulose surface [8, 22]. Porosity of cell wall matrix, an indicative of permeability of cellulases, can be assessed by the solute exclusion [23, 24] and this technique was recently used to correlate sugarcane cell wall pore size with cellulose digestibility after lignin selective removal [25]. However, complete removal of lignin is not required to achieve considerable cellulose hydrolysis yields (i.e., higher than 70%). As a matter of fact, even pretreatments that do not result in lignin removal can generate a substrate with increased cellulose accessibility, provided that lignin relocates in the cell wall matrix [26, 27]. It has also been shown that topochemical distribution of lignin can affect the hydrolysis of sugarcane bagasse cellulose and other grasses [28–30].

Another important limitation in cellulose hydrolysis caused by lignin is unproductive binding. Differently from the barrier effect, lignin binds cellulases (in some cases irreversibly) decreasing the availability of cellulases to catalyze cellulose hydrolysis. This has become more important considering that cellulose hydrolysis at industrial scale is preferably conducted at low enzyme loading, condition at which the unproductive binding of lignin has been demonstrated to be more pronounced [31]. This behavior was observed over a wide range of substrates submitted to several different pretreatments [32–39]. Pretreatments can be designed in order to overcome, or at least partially reduce, the binding effect of lignin. For instance, the negatively sulfonated groups (in the hydrolysis pH) can cause repulsion of the also negatively charged cellulases [40], resulting in reduced unproductive binding [41, 42].

Lignin from different sources binds enzymes to a different extent. For example, wood lignin has higher binding capacity than grass lignin and lignin from the same feedstock can have different protein binding capacities, depending on the pretreatment technology and condition used [33]. Hydrophobicity of lignin and the free phenolic groups content might play a role in the protein (cellulases) binding capacity of lignins [36, 39, 43–45]. On the other hand, the enhancement of lignin hydrophilicity due to the presence of acid groups may result in reduced binding capacity, beneficial for enzymatic hydrolysis of cellulose [4, 46, 47]. In addition, the characteristics of cellulases, such as isoelectric point and hydrophobic surface, can influence the intensity of the lignin–cellulase binding [48, 49].

In order to understand the limiting role of lignin in sugarcane bagasse, an important source of sugars after enzymatic hydrolysis [50], this work first aimed to prepare a range of pretreated sugarcane bagasse (PSCB) samples with varying lignin contents and properties by pretreating bagasse with five thermochemical leading pretreatment methods. These pretreated bagasses were then used to assess their cellulose accessibility and the binding capacity of the lignins after pretreatments in alkaline and acid conditions, with and without the presence of a delignification agent (sulfite ion). Finally, to assess the improved resistance to lignin unproductive binding of a recently developed industrial cellulase preparation, sugar yields of pretreated bagasses hydrolyzed with Celluclast 1.5, a traditional cellulase mixture, and Cellic CTec3 were compared.

## Methods

### Preparation of the pretreated substrates

The feedstock used in this study was sugarcane bagasse obtained from a Brazilian sugarcane mill (approximately 10% moisture content) submitted to five different pretreatments, producing five pretreated sugarcane bagasses. The alkaline pretreatments (NaOH and Na_2_SO_3_) were conducted according to Mendes [14]. In brief, the bagasse was previously impregnated with a NaOH solution in order to achieve a solid:liquid ratio 1:10 and final alkali concentration of 5% (w_NaOH_/w_bagasse_). The bagasse was pretreated for 1 h, at 121 °C, in an autoclave. The same condition was applied for the alkaline sulfite pretreatment, except that Na_2_SO_3_ was added to the NaOH solution, so that the sodium sulfite concentration was 10% (${\text{w}}_{{{\text{Na}}_{ 2} {\text{SO}}_{ 3} }} /{\text{w}}_{\text{bagasse}}$). The acid pretreatments were based on a previous work [47], but adapted to sugarcane bagasse. For the diluted acid pretreatment, the solid:liquid ratio was 1:10 and the H_2_SO_4_ concentration was 0.75% (${\text{w}}_{{{\text{H}}_{ 2} {\text{SO}}_{ 4} }} /{\text{w}}_{\text{bagasse}}$). The soaked bagasse was pretreated in a rotating digester (Aurora Products Ltd, Savona, CA), at 160 °C for 30 min, with a 30-min ramp to reach the temperature. The variation of this pretreatment with sulfite was conducted by adding NaHSO_3_ to the acid solution, resulting in a concentration of 5% (${\text{w}}_{{{\text{NaHSO}}_{ 3} }} /{\text{w}}_{\text{bagasse}}$). Finally, for the SO_2_/steam pretreatment, one of the conditions described by Ferreira-Leitão et al. [51] was used. Prior to the impregnation with SO_2_, the moisture content of the bagasse was increased to 50% in order to make the absorption of the catalyst more efficient. The SO_2_ concentration was 3.0% (${\text{w}}_{{{\text{SO}}_{ 2} }} /{\text{w}}_{\text{bagasse}}$) and the impregnated bagasse was treated in a 2L Stake Tech III steam gun (Stake Technologies, Norvall, CA) at 190 °C for 5 min.

After pretreatment, all solid fractions were washed with tap water (20-fold the dry mass of pretreated bagasse), vacuum-filtered, and frozen until used.

### Chemical composition

The carbohydrate and lignin contents of the raw and pretreated bagasses were determined as previously described [31]. Briefly, the carbohydrates in the substrates were acid hydrolyzed and the sugars were quantified with a Dionex (Sunnyvale, CA) HPLC (ICS-3000). Glucan content was determinate based on the released glucose, and xylan content on the released xylose, arabinose, and acetic acid. The acid insoluble lignin was gravimetrically analyzed and the acid soluble lignin was measured by reading the absorbance at 205 nm, using an extinction coefficient of 105 L g^−1^ cm^−1^ [52].

### Protein concentration and enzyme activities

Protein concentration was determined by a ninhydrin-based method [53]. Filter paper activity of cellulase preparations was measured according to Ghose [54]. β-Glucosidase activity was measured hydrolyzing *p*-nitrophenyl-β-d-glucopyranoside (0.2%) and spectrophotometrically quantifying the released *p*-nitrophenol at 410 nm [55].

### Cellulose digestibility

The washed substrates were enzymatically hydrolyzed using Celluclast 1.5 L (Novozymes, DK) supplemented with β-glucosidase (Novozym 188, Novozymes, DK). Enzymatic hydrolysis was conducted at 2% (w/v) solids in sodium acetate buffer (50 mM, pH 4.8), 50 °C, 120 rpm in a rotatory incubator Combi-H12 (FINEPCR, KR). The total volume was 1.5 mL, in 2.0-mL tubes. The protein loadings, based on the Celluclast protein content (130 mg/mL; 60 FPU/mL), were 2.5, 5, 10, 20, and 30 mg/g of cellulose in the PSCB. Novozym 188 (458 UI/mL) was added in order to have a β-glucosidase activity 2 times higher than the filter paper activity in the reaction mixture. After 72 h, the enzymes were inactivated by heating at 100 °C for 5 min. The glucose concentration in the hydrolysate was measured using the glucose oxidase assay [56]. Hydrolyses were analyzed in triplicates and the standard deviation was calculated.

### Determination of unproductive binding capacity

Each PSCB was hydrolyzed with a combination of Celluclast and Novozym 188 or with Cellic CTec3. However, prior to the addition of the enzymes, the blocking of the lignin binding sites was done according to Kumar et al. [31]. 250 mg of bovine serum albumin (BSA) per g of cellulose was added and incubated for 1 h at 50 °C and 120 rpm. After that, 2.5 mg of protein (based on Celluclast or CTec 3 protein content) per gram of cellulose was added and the hydrolysis was conducted for 72 h. The same hydrolysis condition, but without the prior addition of BSA, was conducted and the difference in hydrolysis yields (with and without BSA) was considered an indication of unproductive binding [31]. Reaction and analysis conditions were the same as described above.

### Estimation of accessible cellulose surface

Accessible cellulose surface was estimated according to Simons [57] and adapted elsewhere [58–61]. Direct Orange (Pontamine Fast Orange 6RN) and Direct Blue (Pontamine Fast Sky Blue 6BX) solutions were prepared at 10 mg/mL. The Direct Orange (DO) solution was ultra-filtered three times in Amicon apparatus with a 100-kDa membrane (filtrations stopped when approximately 80% of the initial volume was eluted). DO concentration was determined by dry weight and re-adjusted to 10 mg/mL. Molar attenuation coefficient of DO was calculated at 624 nm and DB at 455 nm (maximum absorption wavelength of each dye).

Each PSCB (10 mg, dry weight) was added to 2-mL centrifuge tubes, and 0.1 mL of phosphate buffered saline pH 6.0 (PO_4_ 0.3 M, NaCl 1.4 mM) and deionized water were added. The amount of water varied, since the final incubation volume (buffer, water and dye solutions) was 1.0 mL. Prior to the addition of the dye solutions (at 10 mg/mL), the mixture was incubated for 12 h, at approximately 25 °C in the bench. The volumes added were 0.025, 0.050, 0.075, 0.1, 0.15, and 0.2 mL, in each incubation condition. The tubes were vortex mixed and incubated at 70 °C in a water bath, 200 rpm, for 6 h. The mixtures were than centrifuged at 1800 rpm for 5 min and the absorbance of the supernatants was measured in spectrophotometer at 455 and 624 nm. The concentration of each dye was calculated according to the following equations:

$$ \left[ {\text{DO}} \right] = \left[ {\left( {\varepsilon_{\text{DB624}} \times {\text{ A}}_{ 4 5 5} } \right){-}\left( {\varepsilon_{\text{DB455}} \times {\text{ A}}_{ 6 2 4} } \right)} \right]/[\left( {\varepsilon_{\text{DO455}} \times \, \varepsilon_{\text{DB624}} } \right){-}\left( {\varepsilon_{\text{DB455}} \times \, \varepsilon_{\text{DO624}} } \right] $$

$$ \left[ {\text{DB}} \right] = \left[ {\left( {\varepsilon_{\text{DO455}} \times {\text{ A}}_{ 6 2 4} } \right){-}\left( {\varepsilon_{\text{DO624}} \times {\text{ A}}_{ 4 5 5} } \right)} \right]/[\left( {\varepsilon_{\text{DO455}} \times \, \varepsilon_{\text{DB624}} } \right){-}\left( {\varepsilon_{\text{DB455}} \times \, \varepsilon_{\text{DO624}} } \right], $$

where [DO] and [DB] are the concentrations of orange and blue dyes in the solutions, respectively, in mg/mL; ε_DB455_ and ε_DB624_ are the molar attenuation coefficient of the blue dye at 455 and 624 nm, respectively, in L g^−1^ cm^−1^; ε_DO455_ and ε_DO624_ are the molar attenuation coefficient of the orange dye at 455 and 624 nm, respectively. A_455_ and A_624_ are the absorbance values of the solutions at 455 and 624 nm, respectively. The amount of adsorbed dye was calculated subtracting the amount of dye in solution before and after incubation. The amount of adsorbed dye (mg_dye_/g_PSCB_) was plotted against the dye concentration after incubation (mg/mL), for each PSCB. The first plateau was used for the calculation, since Direct dyes can adsorb in double layer [62]. Accessible cellulose surface was estimated as the ratio of maximum amount of adsorbed dyes (DO/DB).

### Quantification of acid groups

The strong and weak acid groups in the pretreated substrates were measured by conductometric titration [63]. In 50-mL centrifuge tubes, 0.1 g of the PSCB was mixed with 1.0 M HCl and left for 12 h at 4 °C. Approximately 30 mL of deionized water was added, vortex mixed, and centrifuged at 5000 rpm for 10 min. This washing procedure was carefully repeated 6 times. After decanting the last washing water, the sample was transferred to a 100-mL beaker and 50 mL of a 0.001 M NaCL solution was added. The suspension was constantly mixed with a magnetic stirring bar and 20 µL of 0.5 M HCl solution was added. The mixture was titrated with 0.05 M NaOH, recording the conductivity of the mixture after each addition of the alkali solution, at 10 µL increments. The decrease in the conductivity after neutralization of the HCL in solution (by the addition of 200 µL of NaOH solution) was associated with the presence of strong acid groups in the substrate. The next phase of the titration curve, in which the conductivity did not change with the addition of sodium hydroxide, was associated with the presence of weak acid groups. After the neutralization of the acid groups, the conductivity increased with the addition of NaOH. To calculate the amount of acid groups in the substrates, the number of moles of NaOH required to neutralize the strong and weak acid groups in the substrate was divided by the mass of PSCB (weak and total acid groups) or lignin in the PSCB (strong acid groups).

### Lignin isolation

The lignin isolation procedure was adapted from two methodologies [64, 65]. The PSCB (30 g, dry weight) was hydrolyzed with Celluclast 1.5L (65 FPU/g cellulose), β-glucosidase (120 UI/g cellulose), and Cellic HTec2 (Novozymes, DK; 20 mg protein/g cellulose), for 72 h. The mixture was centrifuged (5000 rpm, 60 min), the supernatant decanted, and the solids washed with 700 mL of distilled water. The residual solid was suspended in 400 mL of a 50 mM phosphate buffer solution (pH 7) containing 0.2 U/mL Protease type XIV (Sigma, US), 40 mg/L tetracycline, and 30 mg/L cycloheximide and the mixture was incubated for 12 h at 37 °C, 120 rpm. The residue was washed with 800 mL of 1 M NaCl, followed by 1.6 L of distilled water to remove residual carbohydrates and proteins, and then filtered. The solid was air-dried and ground in a Willey Mill to 60 mesh.

The lignin in the ground residue was solvent extracted with acidified aqueous dioxane solution (dioxane/water 85:15, v/v, containing 0.01 M HCl). In a distillation flask connected to a condenser, 200 mL of the solution was added to 10 g of ground residue. The suspension was mixed with a magnetic stirrer and heated (boiling point of the azeotrope, 86 °C) under argon atmosphere, for 2 h. The resulting suspension was filtered; the filtrated was neutralized with sodium carbonate and evaporated in a rotatory evaporator in order to reduce the volume. The solution was added dropwise to 1 L of acidified deionized water (pH 2), constantly mixing. The precipitated lignin was allowed to equilibrate with the aqueous phase for 12 h, recovered by centrifugation, washed with 800 mL of water, and freeze-dried.

### Adsorption capacity of the isolated lignin

The adsorption capacity of the isolated lignins was measured by adding cellulases solution (diluted Celluclast) at different concentrations to the same amount of lignin [35]. In 2 mL centrifuge tubes, 1.0 mL of the cellulase solution (from 0.05 to 0.4 mg/mL) was added to 20 mg of lignin. The tubes were incubated at 50 °C in, 120 rpm, to simulate the hydrolysis conditions. After 3 h, the mixture was centrifuged at 10,000 rpm for 10 min and the protein concentration in the supernatant was measured by the ninhydrin assay [53]. The amount of protein adsorbed to the lignin was the difference between the protein concentration of the added cellulase solution and the supernatant after incubation. The amount of protein bound to the lignin (mg/g of lignin) was plotted against the concentration of protein in the supernatant (mg/mL). Data were fitted in a Langmuir model, using the software OriginPro 9.0.

## Results and discussion

### Pretreatments and chemical characteristics of the sugarcane bagasses

Sugarcane bagasse (SCB) was pretreated in alkaline and acidic conditions aiming to prepare varied substrates presenting similar glucan contents, but different levels of residual xylan and lignin. Chemical composition of PSCBs and removal of cell wall components are presented in Table 1. Cellulose content of the pretreated bagasses was similar, varying from 50.5% (NaOH PSCB) to 56.1% (SO_2_/steam PSCB). As expected, cellulosic fraction was more preserved in alkaline conditions (less than 2.5% solubilization), contrasting with 13–22% solubilization in acidic pretreatments. Solubilization of the hemicellulosic fraction was also greater in acid conditions, since hydrolysis reactions of this component are favored at low pH. Xylan content of H_2_SO_4_ PSCB, NaHSO_3_/H_2_SO_4_ PSCB, and SO_2_/steam PSCB was, respectively, 13.8, 10.1, and 2.0%, whereas in NaOH PSCB and Na_2_SO_3_/NaOH PSCB, the xylan content was 25.6 and 26.1%, respectively.

**Table 1:** Chemical composition (%) of pretreated sugarcane bagasses, solids yields, and removal of cell wall components during the pretreatments

Pretreatment	Chemical composition (g/100 g of pretreated material)			Solids yields (%)	Removal of cell wall components after pretreatment (%)		
	Glucan	Xylan	Lignin		Glucan	Xylan	Lignin
Untreated bagasse^a^	44.8 (1.0)	25.5 (0.4)	24.4 (0.7)	–	–	–	–
NaOH (5%)	50.5 (0.8)	25.6 (0.3)	20.3 (0.5)	87.3	0.0	19.4	26.3
Na_2_SO_3_/NaOH (10%/5%)	52.2 (0.6)	26.1 (0.4)	15.0 (0.7)	81.8	2.3	22.7	48.8
H_2_SO_4_ (0.75%)	53.1 (1.0)	13.8 (0.3)	28.8 (1.1)	71.1	13.5	64.1	14.6
NaHSO_3_/H_2_SO_4_ (9%/0.75%)	54.0 (0.3)	10.1 (0.1)	28.3 (0.6)	63.3	21.7	76.7	25.4
Steam explosion/SO_2_ (3.0%)	56.1 (0.6)	2.0 (0)	35.6 (0.8)	60.8	22.0	95.8	10.0

Values in parentheses are standard deviations

^a^ Untreated bagasse contained 4.5% of water/ethanol-soluble extractives. Pretreated materials were analyzed without previous extraction

Alkaline and acidic conditions were tested specially because of the differences in lignin solubilization. At high pH values, lignin solubilization is facilitated, and it is enhanced by the presence of sulfite ions. On the other hand, in acid conditions, lignin can polymerize, decreasing its solubility. The presence of hydrogen sulfite in acidic reactions aids lignin solubilization, preventing polymerization [41, 66]. Lignin solubilization reached 26.3 and 48.8% after NaOH and Na_2_SO_3_/NaOH pretreatments, respectively. Less lignin was solubilized during 3.0% SO_2_/steam and H_2_SO_4_ pretreatments, representing 10 and 14.6%, respectively. The higher concentration of hydrogen sulfite ions present in the 9%-NaHSO_3_/H_2_SO_4_ pretreatment enhanced lignin solubilization (25.4%) to a value similar to NaOH pretreatment (26.3%). As a consequence of these different delignification levels, PSCB with contrasting lignin contents was generated, ranging from 15.0% (Na_2_SO_3_/NaOH PSCB) to 35.6% (SO_2_/steam PSCB) (Table 1). This difference was important to assess the effect of lignin in limiting the enzymatic hydrolysis of PSCB.

**Fig. 1:**
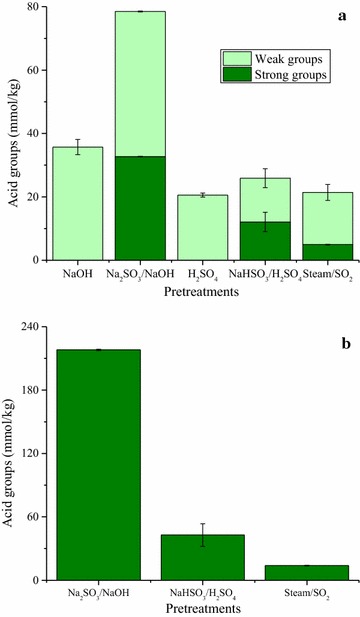
Strong and weak acid groups in the PSCB (**a**) and in the lignin (**b**). Only the amount of strong acid groups was divided by the amount of lignin in each substrate

Pretreatments can create, expose, or even remove acid groups in lignocellulosic materials structure, which can greatly influence the subsequent enzymatic hydrolysis of polysaccharides in pretreated materials [41]. The presence of weak acids in polysaccharides (glucuronic acid for example) and also in lignin is attributed to carboxylic groups. Sulfonic acid groups are considered strong acids [63]. Total acid groups content, determined by conductometric titration (Fig. 1), varied among the PSCB. For the Na_2_SO_3_/NaOH and NaOH PSCB, it was found 78.5 and 35.7 mmols of acid groups per kg of PSCB, respectively. Lower values were detected under acidic conditions. The presence of strong acid groups can be considered a consequence of lignin sulfonation [67]. Therefore, the current results indicate strong sulfonation of residual lignin contained in the Na_2_SO_3_/NaOH PSCB (218.0 mmol/kg of lignin), whereas the hydrogen sulfite pretreatments (NaHSO_3_/H_2_SO_4_ and SO_2_/steam) resulted in moderate to low sulfonation degrees of PSCBs (42.8 mmol/kg of lignin and 13.9 mmol/kg of lignin, respectively) (Fig. 4b). In the Na_2_SO_3_/NaOH and NaHSO_3_/H_2_SO_4_ pretreatments, sulfite ions promoted not only greater lignin solubilization (Table 1), but also stronger sulfonation of the residual lignin (Fig. 1).

### Enzymatic digestibility of the pretreated SCB

**Fig. 2:**
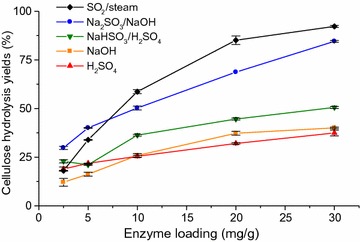
PSCB cellulose hydrolysis yields after 72 h with Celluclast and Novozym 188, at varying enzyme loadings and 2% (w/w) solid loading

Determination of cellulose hydrolysis yields using different enzyme loadings provides information about substrate digestibility. Therefore, pretreated SCB was enzymatically hydrolyzed using Celluclast (cellulases) and Novozym 188 (source of β-glucosidase) (Fig. 2). Enzyme loading, based on protein concentration, ranged from 2.5 to 30 mg/g (protein/g of cellulose) and was calculated based only on Celluclast protein concentration. The glucan hydrolysis yields of NaOH, NaHSO_3_/H_2_SO_4_, and H_2_SO_4_ PSCB were lower than 50%, even at the highest enzyme loading tested (30 mg/g). Moreover, the hydrolysis yields of these substrates were only slightly enhanced with increased enzyme loading (Fig. 2). On the other hand, 82–92% of the cellulose in the Na_2_SO_3_/NaOH PSCB and SO_2_/steam PSCB was hydrolyzed, respectively, at the highest enzyme loading (30 mg/g). The hydrolysis yields of these substrates were also significantly dependent on the enzyme loading.

This experimental enzymatic hydrolysis approach enabled the comparison of the accessibility of cellulose in SCB submitted to different pretreatments. That is, if the increase in enzyme loading does not result in significant enhancement of cellulose hydrolysis yields, enzyme access to cellulose molecules are most likely limited due to the presence of lignin and/or hemicellulose (considering that the excess of enzymes could overcome any inhibitory effect). On the other hand, accessible cellulose is likely to be more intensively digested with increasing enzyme loadings [31]. Among the currently evaluated substrates, SO_2_/steam, and Na_2_SO_3_/NaOH PSCBs were the least recalcitrant, reaching almost complete cellulose hydrolysis with the highest enzyme loading. This may indicate that the cellulose in these substrates was more accessible to the enzymes than in the other substrates. However, this can be controversial if lignin and hemicellulose contents are taken into account to explain the effect of accessibility of cellulose in the hydrolysis yields. The current data with PSCBs clearly indicated that two significantly different substrates (low xylan and high lignin contents in SO_2_/steam PSCB, and high xylan and moderate lignin contents in Na_2_SO_3_/NaOH PSCB) presented similar digestibilities. Clearly, additional and multi-correlated characteristics of the substrates are defining their digestibilities. For example, lignin was not removed during SO_2_/steam pretreatment but lignin relocation can be a key factor in increasing cellulose accessibility [66]. Associated with extensive xylan removal, this might explain the low recalcitrance of SO_2_/steam PSCB, even though it had the highest lignin content. Rollin et al. [9] also observed that a pretreated switchgrass with relatively high lignin content was readily digested by cellulases. They emphasized that increasing cellulose accessibility is a key factor to reach effective enzymatic hydrolysis of lignocellulosic biomasses, even more important that lignin removal. In contrast, the Na_2_SO_3_/NaOH PSCB, despite presenting moderate lignin and xylan removals, contained a significantly sulfonated residual lignin, which could have contributed to a more hydrophilic and swollen substrate with high digestibility [20].

Another observation worth mentioning is the behavior of the two most digestible substrates (Na_2_SO_3_/NaOH and SO_2_/steam PSCB) under low enzyme loadings. Even though the hydrolysis yields of SO_2_/steam PSCB was higher than of Na_2_SO_3_/NaOH PSCB at enzyme loadings higher than 10 mg/g, at lower enzyme loadings (2.5 and 5 mg/g) that approximates desirable conditions in economically viable processes [68], the Na_2_SO_3_/NaOH PSCB cellulose was more hydrolyzed than the former PSCB. Different experimental approaches were then conducted in order to better understand this distinct behavior and comprehend the limiting role of lignin in the enzymatic hydrolysis of cellulose in the PSCB.

### Effect of unproductive binding

**Fig. 3:**
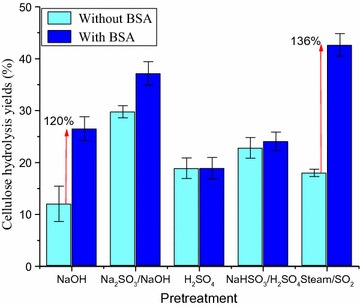
PSCB cellulose hydrolysis yields after 72 h hydrolysis with Celluclast (2.5 mg/g) and Novozym 188 (2.3 UI/g), at 2% (w/w) solid loading. Experiments were run with and without 1 h-preincubation of the PSCB with BSA (250 mg/g of cellulose)

An important limiting effect of lignin, especially at low enzyme loadings, is unproductive binding. Since the hydrolysis yields seemed to be dictated by cellulose accessibility at enzyme loadings higher than 5 mg/g, the effect of unproductive binding at 2.5 mg/g was investigated. To block the lignin unproductive binding sites, BSA was added to hydrolysis media 1 h before the addition of enzymes [31]. Hydrolysis yields of PSCB cellulose with and without addition of BSA are compared in Fig. 3.

Addition of BSA enhanced the cellulose hydrolysis yield of NaOH, Na_2_SO_3_/NaOH, and SO_2_/steam PSCB. The enhancement was more pronounced in SO_2_/steam PSCB (120%) and NaOH PSCB (136%). This result suggests that the low cellulose hydrolysis yields of these PSCB at low enzyme loadings could be assigned to unproductive binding of cellulolytic enzymes to the poorly charged (sulfonated) lignin (Fig. 3). Although an enhancement of hydrolysis yield was also observed in the hydrolysis of Na_2_SO_3_/NaOH PSCB (25%), it was much lower than in the aforementioned substrates, indicating that even though the pretreatment was conducted in alkaline conditions as in NaOH pretreatment, the effective sulfonation of Na_2_SO_3_/NaOH PSCB lignin may have reduced lignin negative binding influence. Sulfonated lignins bind less cellulases at hydrolysis conditions due to repulsive forces (negatively charged sulfonic groups and some of the cellulases), reducing unproductive binding [41].

### Cellulose accessibility versus unproductive binding in lignin

The accessible cellulose surface was assessed through a colorimetric technique developed by Simons [54] and adapted by several authors [58–61]. The methodology is based on the combination of two dyes, direct orange (DO) and direct blue (DB), and their distinct affinity for cellulose and ability to diffuse within the substrate. DO displays higher affinity for cellulose and presents hydrodynamic radius comparable to some cellulases, which means that the orange dye may be used to estimate the accessibility of cellulose to cellulases. On the other hand, DB has lower affinity for cellulose, but is smaller than DO, allowing it to reach surfaces that DO (and consequently cellulases) cannot access [61]. By measuring the ratio of the amount of adsorbed dyes (DO/DB), it is possible to obtain some information about the accessible cellulose surface in each PSCB [69]. The amount of adsorbed dyes, as well their ratio, is shown in Table 2. The most recalcitrant substrates (NaOH, H_2_SO_4_, and NaHSO_3_/H_2_SO_4_ PSCB) adsorbed more DB than DO, indicating that the majority of the pores that give access to cellulose fibrils in these substrates were smaller than the size of the orange dye. The less recalcitrant substrates (Na_2_SO_3_/NaOH and SO_2_/steam PSCB) adsorbed more DO than DB, confirming that the cellulose was more exposed (accessible) in these substrates.

**Table 2:** Adsorbed direct orange (DO) and direct blue (DB) on PSCB after 6 h incubation at 70 °C and the ratio between them (accessible cellulose surface)

Pretreatment	Adsorbed DO (mg/g)	Adsorbed DB (mg/g)	DO/DB
NaOH	18.35	26.41	0.69
Na_2_SO_3_/NaOH	24.27	21.19	1.14
H_2_SO_4_	17.38	24.9	0.69
NaHSO_3_/H_2_SO_4_	19.84	25.43	0.78
SO_2_/steam	23.92	22.65	1.05

As expected, greater DO/DB values were observed in substrates with more accessible cellulose surface (higher DO adsorption), making them more easily hydrolyzed by cellulases. In fact, the more enzymatically digestible substrates showed higher DO/DB values (1.14 in Na_2_SO_3_/NaOH PSCB and 1.05 in SO_2_/steam PSCB). In the least digestible substrates NaOH, H_2_SO_4_, and NaHSO_3_/H_2_SO_4_ PSCB, DO/DB ratio was lower than 0.78.

**Fig. 4:**
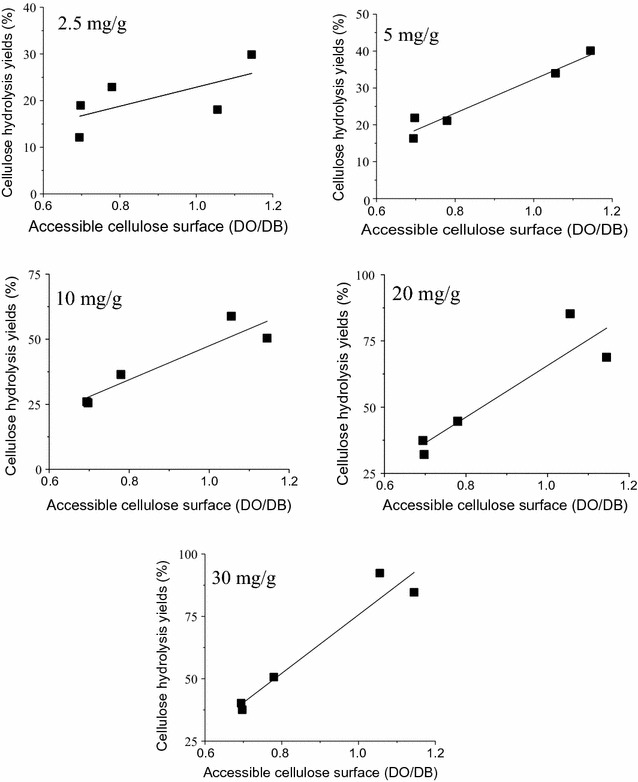
Correlation between PSCB cellulose hydrolysis yields after 72 h with Celluclast and Novozym 188 and the accessible cellulose surface. NaOH PSCB (*orange*), H_2_SO_4_ PSCB (*red*), NaHSO_3_/H_2_SO_4_ PSCB (*green*), SO_2_/steam PSCB (*black*), and Na_2_SO_3_/NaOH PSCB (*blue*). Enzyme loadings were 2.5, 5, 10, 20, and 30 mg/g

In order to correlate the cellulose accessibility of the substrates with the enzymatic hydrolysis efficiency, DO/DB ratio was plotted with the cellulose hydrolysis yields after 72 h of hydrolysis at increasing enzyme loadings (Fig. 2). Except for the lowest enzyme loading (2.5 mg/g, Fig. 4), hydrolysis yields showed good correlation with accessible cellulose (DO/DB), with R^2^ values higher than 0.83. Similar correlation values were also reported by Arantes and Saddler [69] for a range of substrates pretreated by different pretreatment technologies. The cellulose in Na_2_SO_3_/NaOH and SO_2_/steam PSCB was more accessible and this is reflected in the digestibility of these substrates. Na_2_SO_3_/NaOH treatment resulted in the highest lignin solubilization (Table 1), which might have contributed to increase cellulose accessibility. On the other hand, even though SO_2_/steam PSCB had the lowest lignin solubilization and consequently the highest lignin content of all the PSCB, cellulose accessibility was comparable to Na_2_SO_3_/NaOH PSCB probably because the xylan removal was almost complete and also because the steam explosion pretreatment can relocate lignin, which may have also contributed to increase cellulose accessibility [26, 70].

**Fig. 5:**
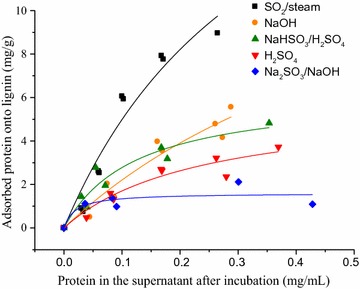
Langmuir adsorption isotherms of Celluclast proteins onto isolated lignins. Lignin concentration was kept constant (20 mg/mL) and initial protein concentration varied from 0.05 to 0.45 mg/mL

With the lowest enzyme loading tested, desirable condition in economically viable processes [68], *R* ^2^ value was 0.43. It has been broadly reported that, besides limiting the access of the enzymes to the cellulose surface, lignin is known to adsorb proteins. Kumar et al. [31] showed that unproductive binding of cellulases to lignin is more pronounced at low enzyme loading, and that this effect could be totally overcome by increasing the enzyme loading. Apparently the binding capacity of softwood lignin, presented in the cited work, has a higher binding capacity than sugarcane bagasse lignin, since at slightly higher enzyme loading (i.e., 5 mg/g), good correlations could be observed. To test the hypothesis that unproductive binding was limiting the hydrolysis at low enzyme loading, and consequently resulting in this poor correlation, the binding capacities of the lignins isolated from PSCB bagasse were measured. Lignin samples isolated from the PSCB were incubated in increasing concentrations of protein (Celluclast), keeping lignin concentration constant. The adsorption curves are shown in Fig. 5 and the constants extracted from Langmuir adsorption model are presented in Table 3.

**Table 3:** Parameters from the adsorption isotherm of the proteins from Celluclast onto lignins extracted from the PSCB

Pretreatment	*P*_max_ (mg/g)	*K* (mL/mg)	*R* ^2^
NaOH	17.0 ± 11.2	1.5 ± 0.3	0.93
Na_2_SO_3_/NaOH	1.6 ± 0.4	50.8 ± 70.3	0.63
H_2_SO_4_	6.2 ± 1.1	8.0 ± 3.2	0.90
NaHSO_3_/H_2_SO_4_	5.8 ± 1.5	4.2 ± 2.1	0.91
SO_2_/steam	23.2 ± 8.7	2.7 ± 1.5	0.93

Values in parentheses are standard deviations

Maximum protein binding capacity of lignin samples ranged from 1.6 (Na_2_SO_3_/NaOH PSCB) to 23.2 (SO_2_/steam PSCB) mg of protein per gram of lignin. Comparing all the lignin samples, it was possible to observe that lignin sulfonation extent was a key factor in reducing the protein binding capacity of lignin. Under alkaline and acidic conditions, the presence of sulfite ions during the pretreatment results not only in enhanced lignin solubilization, but also in sulfonation of the lignin that remains in the solid fraction (Fig. 1). Sulfonic groups present in the residual lignin are strong acids and are deprotonated (negatively charged) in the experimental conditions (pH 4.8) described in this work [41]. Most of the cellulases from *T. reesei* extract (as Celluclast) have isoelectric point lower than this pH value [48] and consequently are also negatively charged in this condition. Therefore, the negative charge of sulfonated lignin can promote repulsion of cellulases, reducing the binding capacity of lignin. In fact, as a result of the addition of sulfite ions to alkaline pretreatment, the binding capacity of residual lignin reduced from 17.0 mg/g (NaOH PSCB) to 1.6 mg/g (Na_2_SO_3_/NaOH PSCB). Under acidic conditions, the difference was not that evident, but it was still possible to observe the reduction in the protein binding capacity of lignin, from 6.2 mg/g (H_2_SO_4_ PSCB lignin) to 5.8 mg/g (NaHSO_3_/H_2_SO_4_ PSCB lignin). Although strong acid groups were detected in SO_2_/steam PSCB (Fig. 4b), the sulfonation of this lignin was very low, resulting in a strong binding capacity.

### Effect of BSA addition in the enzymatic hydrolysis of PSCB using different cocktails

**Fig. 6:**
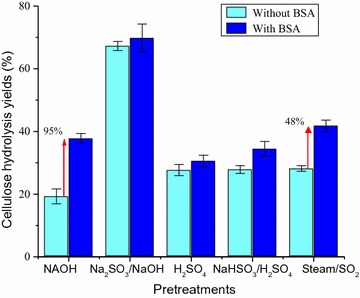
PSCB cellulose hydrolysis yields after 72 h hydrolysis with Cellic CTec3 (2.5 mg/g), at 2% solids loading. Experiments were run with and without preincubation of the PSCB with BSA

As discussed earlier, unproductive binding depends not only on the lignin properties, but also on enzyme characteristics. Berlin and colleagues [32] pointed out the importance of enzyme development in the concept of weak lignin-binding enzymes. For that reason, the effect of unproductive binding was also tested in a more advanced enzyme preparation, Cellic CTec3 (Fig. 6), an industrial cellulase cocktail recently designed and optimized specifically for enzymatic hydrolysis of pretreated lignocellulosic substrates. With Celluclast, unproductive binding was observed for NaOH, Na_2_SO_3_/NaOH, and SO_2_/steam PSCB (Fig. 3). With CTec3, no significant enhancement in hydrolysis yield due to BSA addition was observed in Na_2_SO_3_/NaOH PSCB, indicating no unproductive binding during the hydrolysis of this substrate with CTec3. Moreover, an impressive 70% cellulose hydrolysis yield was achieved with a considerable low enzyme loading (2.5 mg/g), probably as a result of more efficient enzyme combination (cellulases and accessory enzymes) that efficiently deconstructs substrates with high hemicellulose content. For SO_2_/steam PSCB hydrolyzed with CTec 3, a 48% increase in hydrolysis yield was observed due to BSA addition (Fig. 6), whereas with Celluclast, the increment was 136% (Fig. 3). A lower increase was also observed with NaOH PSCB (120% with Celluclast and 95% with CTec3, Figs. 3, 6, respectively). The lower increase in hydrolysis yields in these substrates with high protein binding capacity lignins is an indication of higher tolerance of CTec3 to the presence of lignin.

## Conclusions

Pretreatments that efficiently removed (Na_2_SO_3_/NaOH) or relocated (SO_2_/steam pretreatment) lignin resulted in substrates with greater cellulose accessibility and, consequently, were more efficiently digested by cellulases. Substrates containing lignin with high protein binding capacity resulted in high unproductivity binding at low dosage of traditional cellulases as observed during hydrolysis of NaOH PSCB and SO_2_/steam PSCB with Celluclast. Pretreatments that promoted sulfonation of lignin reduced its protein binding capacity as a consequence of the negative charges. In addition, a recently developed cellulose preparation, Cellic CTec3, was shown to be less affected by the unproductive binding capacity of lignin, and promoted higher hydrolysis yield, even at very low enzyme loading. These results confirm the importance of pretreatments that increase cellulose accessibility and modify the lignin in order to decrease its protein binding capacity. Also, considering that the enzymatic hydrolysis in industrial processes must be conducted at low enzyme loadings, Na_2_SO_3_/NaOH pretreatment might be promising, since the results show that an impressive amount of cellulose (70%) was hydrolyzed with low enzyme loadings such as 2.5 mg of protein per gram of cellulose with the modern enzyme preparation Cellic CTec3.
